# Does Neutral Affect Exist? How Challenging Three Beliefs About Neutral Affect Can Advance Affective Research

**DOI:** 10.3389/fpsyg.2019.02476

**Published:** 2019-11-08

**Authors:** Karen Gasper, Lauren A. Spencer, Danfei Hu

**Affiliations:** Department of Psychology, The Pennsylvania State University, University Park, PA, United States

**Keywords:** neutral affect, belief, emotion, affect-as-information, valence

## Abstract

Researchers interested in affect have often questioned the existence of neutral affective states. In this paper, we review and challenge three beliefs that researchers might hold about neutral affect. These beliefs are: (1) it is not possible to feel neutral because people are always feeling something, (2) neutrality is not an affective state because affect must be positively or negatively valenced, and (3) neutral affect is unimportant because it does not influence cognition or behavior. We review the reasons these beliefs might exist and provide empirical evidence that questions them. Specifically, we argue that neutral affect is a felt experience that provides important valence-relevant information, which influences cognition and behavior. By dispelling these beliefs about neutral affect, we hope to shine a light on the assumptions that researchers hold about the nature of affect and to provide novel theoretical and methodological perspectives that help advance our understanding of the affective landscape.

## Introduction

Does neutral affect exist? Researchers interested in affect often do not consider this question because they are focused on understanding the presence of feelings, not the presumed lack of them. Yet, how researchers think about the possibility of a neutral affective state is an interesting exercise because it exposes researchers’ fundamental beliefs concerning the nature of affect. This paper challenges three beliefs that researchers might have about the existence of neutral affect. For each belief, we present evidence that questions it and provide a new way to think about the nature of affect. In the last section of the paper, we discuss how these new perspectives could potentially lead to theoretical and methodological innovations.

Prior to discussing what beliefs researchers might hold about whether neutral affect exists, it is necessary to define some terms. Affect is defined as a feeling state (Schimmack and Crites, 2005; Barrett and Bliss-Moreau, 2009). Traditionally, affect possesses at least two key qualities: valence (pleasantness/unpleasantness) and arousal (Wundt, 1897; Clore and Schnall, 2005; Barrett and Bliss-Moreau, 2009). People might experience their affective states as reactions to whatever they are currently thinking about (Clore and Schnall, 2005). For example, positive or negative affect might be experienced as positive or negative evaluations about an object, a person, or a topic (Clore and Schnall, 2005). People also can experience affect as a quality or feature of the stimulus itself, such as when one views a roller coaster as scary. However, as Barrett and Bliss-Moreau (2009) pointed out, what makes the coaster seem scary is not the coaster *per se*, but rather people’s experience of it. That is, affect is still being experienced as an indicator of people’s own evaluative reactions to the world. Affect is a general term that encompasses affective traits, moods, and emotions. Moods and emotions are both affective states. Moods, however, are generally less intense states and longer in duration than emotions. Also, unlike emotions, which often have a clear object (e.g., I am anxious when I see a snake), moods typically lack a salient cause (e.g., I woke up feeling a bit anxious; Ekman and Davidson, 1994; Beedie et al., 2005).

We define neutral affect as *feeling* indifferent, nothing in particular, and a lack of preference one way or the other. Note, when we use the term “indifferent,” we do not use it to indicate disliking something because that would imply a negative rather than a neutral reaction. It also is important to keep in mind that neutral affect could, theoretically, co-occur with positive and/or negative affect^1^. For example, a parent might be relaxing on the sofa when their child asks if they can go play together in the park. The parent might feel neutral about the prospect, in that they did not particularly want to go to the park, but they also are not against going to the park. Even though the parent feels neutral about going to the park, the parent also might feel happy because their child wants to spend time with them. Neutral affect is thus defined as the presence of neutral affect rather than the absence of, or low levels of, positive and negative affect. In the next sections, we provide additional details about neutrality, including how to conceptualize and measure it.

In this paper, we examine three key beliefs that researchers might hold about the nature of neutral affect. These beliefs are: (1) it is not possible to feel neutral because people are always feeling something; (2) neutrality is not an affective state, because affect must be positively or negatively valenced; and (3) neutral affect is unimportant because it does not influence cognition or behavior. In the process of discussing evidence that questions these beliefs, we shed light on researchers’ assumptions about what affect is and provide alternative perspectives that could lead to theoretical and methodological discoveries.

## Belief: It is not Possible to Feel Neutral Because People are Always Feeling Something

The first belief we want to discuss is the notion that neutral affect does not exist because people are always feeling something (Damasio, 2003; Izard, 2007). Damasio (2003) pointed out that, “… all of your experiences occur in an emotion-full world. The point is, we do not live in a neutral world. Our experiences are always emotionally loaded…” (p. 50). Izard (2007) stated, “…there is no such thing as an affectless mind; affect or emotion is always present” (p. 270). Helson (1964) wrote, “All experience is more or less tinged with affect” (p. 341). Because affect is always present, some researchers might believe that it is impossible for people to feel nothing. Therefore, neutral affect does not exist.

A weaker version of this belief is that neutral affect might occur, but it is a rare or fleeting occurrence. For instance, Wundt (1897) acknowledged the existence of neutral affect, but as a rare occurrence, stating “… we are perhaps never in a state entirely free from feeling, although the general nature of feelings demands an indifference-zone.” Similarly, Tomkins and McCarter (1964) wrote, “… it is more common for human beings to feel affect than to feel no affect” (p. 150). Brendl and Higgins (1996) reviewed work indicating that the absence of positive is experienced as negative; whereas the absence of negative is experienced as positive. If so, perhaps neutrality might rarely occur. Other researchers question whether neutral moods can ever be created in the lab because what typically is considered a neutral mood often still is valenced. Indeed, Forgas (1999) noted that “…it is not possible to induce experimentally a genuinely neutral mood in participants” (p. 933). Other researchers have called neutral mood manipulations a “misnomer” (Albarracin and Hart, 2011) or a “so-called neutral mood” (Gendolla, 2012), reflecting their skepticism about them.

The belief that neutral affect does not exist or that it is a very rare occurrence might stem from unwarranted assumptions about the nature of neutral affect. First, the belief that neutral affect does not exist because we are always feeling something often stems from the assumption that neutral affect reflects the literal absence of feeling. Indeed, researchers have described their neutral affect conditions as non-affective conditions in which no emotion is elicited (Fredrickson, 1998; Rotteveel et al., 2001; Evers et al., 2009). But what if feeling neutral is not the literal absence of affect, but rather the presence of neutral affect? We argue that neutral affect is not akin to literally feeling nothing, but rather akin to *feeling nothing in particular*.

Second, some researchers assume that if any valenced state is present, then a person is not neutral. Part of the problem with this all-or-none assumption is that people often feel multiple states at once (Roseman, 2011). For instance, even after undergoing a sad mood manipulation, people report experiencing some happiness (Samson et al., 2016). The happiness does not negate the feelings of sadness. Similarly, it might be possible for people to view their experience as neutral, but still report the presence of other affective states.

A third reason why, at first blush, researchers might think that neutral affect does not exist or rarely occurs is that, at least in English, one can easily imagine people saying that they feel “happy,” “sad,” “mad,” and “anxious,” but not “neutral.” The availability heuristic suggests that if an idea does not easily come to mind, we think it is less likely (Tversky and Kahneman, 1973). Indeed, Watson et al. (1999) argued that one of the reasons they view an activation/arousal dimension within mood research as problematic is that “…it has proven difficult to identify affectively neutral terms that fall directly on the hypothesized Activation axis” (p. 829). The assumption is that if people do not use neutral terms to describe their feelings, then neutral states are neither a common nor important occurrence. Failure to identify appropriate neutral terms might not be indicative of this state rarely occurring, but rather a function of researchers not selecting appropriate terms in the English language. English does have terms to reflect neutral affect, such as when people say that they feel “meh,” “so-so,” or “nothing in particular.” For affectively based evaluations, when people feel neutral about issues, they might say, “I am neutral,” but they also say things like “whatever,” “it’s all the same to me,” or “I don’t have a preference.” People express neutral emotions *via* their facial expressions and even when texting *via* neutral face emojis. Researchers’ failure to identify neutral states might stem from them not appropriately assessing it.

A fourth reason why some researchers might not consider neutral affect to be a commonly experienced state stems from culture. Eastern cultures place more importance on affective balance than Western cultures (Sims et al., 2015). This emphasis on balance might be because East Asian cultures, especially those influenced by Confucianism, tend to value balance, moderation, equilibrium, and seeking the “middle-way” (Peng and Nisbett, 1999). If so, neutral affect might be more common in Eastern than Western cultures because it can reflect viewing one’s self as feeling nothing in particular. Consistent with this hypothesis, Mesquita and Karasawa (2002) asked American and Japanese college students to complete an emotion questionnaire four times a day. The first question was whether the person had experienced an emotion in the past 3 h. The percentage of students in the American sample and in the Japanese students studying in America that selected that they had not experienced any emotion was 7.5 and 6.67%, respectively. Yet, the percentage increased to 22% when one looked at Japanese students who were studying in Japan. It is important to keep in mind that saying one did not experience an emotion is not necessarily akin to saying that one felt neutral, but it does confirm that cultural expectations might guide the notion that one must feel some emotion rather than no emotion in particular. Thus, cultural practices might result in Western participants reporting less neutral affect compared to some Eastern cultures.

But what is the empirical evidence that people do indeed feel neutral affect? One way to gather these data is to ask respondents to rate the intensity of their neutral feelings. Although this practice is not yet commonplace, when researchers engaged in it, respondents reported experiencing neutral reactions (Storm and Storm, 1987; Zelenski and Larsen, 2000; Tay, 2011; Gasper and Hackenbracht, 2015; Gasper and Danube, 2016; Samson et al., 2016; Gallegos and Gasper, 2018). In general, these data indicated that respondents experience neutral states often and at levels on par with or just below experiences of positive affect (Zelenski and Larsen, 2000; Gallegos and Gasper, 2018). For instance, Gallegos and Gasper (2018) examined whether being socially rejected, accepted, or neither influenced people’s feelings of neutrality (assessed by the extent to which people felt indifferent, nothing, emotionless, so-so, did not feel strongly one way or the other, and meh) on a scale ranging from 1 (*not at all*) to 7 (*extremely*). In the control conditions, mean levels of neutral affect (Experiments 1 to 3, respectively: *M* = 3.50, SD = 1.42; *M* = 3.02, SD = 1.12; *M* = 3.05, SD = 1.33) were greater than or not statistically different from mean levels of positive affect (*M* = 2.69, SD = 1.24; *M* = 3.31, SD = 1.15; *M* = 3.31, SD = 1.36; *t*(54) = 3.06, *p* = 0.003; *t*(57) = 1.66, *p* = 0.10; *t*(186) = 1.68, *p* = 0.10). Moreover, if neutral affect does not exist, then it would not be possible to experimentally induce it in the lab. Yet, the few studies that examined the effectiveness of neutral mood manipulations by directly assessing neutral affect indicate that respondents do indeed report more neutral affect than positive or negative affect after viewing stimuli designed to create neutral moods, such as neutral photos (Gasper and Hackenbracht, 2015) and videos (Samson et al., 2016). Thus, people report experiencing neutral affect at relatively high levels, and it is possible to induce neutral affective states. These data clearly question the belief that neutral affect never or rarely occurs.

Even if neutral affect is frequently reported, what is the evidence that neutral affect is a *felt* experience? Perhaps neutral affect reflects feeling nothing and hence there is no experience. One way to examine whether neutral affect is a felt experience is to investigate to what extent neutral affective reactions occupy working memory capacity. Theories such as the absorption hypothesis (Erber and Tesser, 1992) and the mere resource hypothesis (Van Dillen and Koole, 2007) argue that affective states create affect-related thoughts. These thoughts occupy working memory capacity. If affect needs working memory capacity to be experienced, then the intensity of felt affective reactions could be reduced by asking people to engage in cognitively demanding tasks that would compete for these mental resources (Erber and Tesser, 1992; Rusting and Nolen-Hoeksema, 1998; Gerin et al., 2006; Joormann et al., 2007; Van Dillen and Koole, 2007; Kron et al., 2010). Gasper and Hackenbracht (2015) hypothesized that if neutral affect is felt, then it too should be experienced at a lower intensity when working memory capacity is taxed than when it is not. To test this idea, they asked respondents to view positive, negative, or neutral images, to complete either a cognitively demanding or nondemanding task, and then to rate their positive, negative, and neutral states. Consistent with the hypothesis that neutral affect occupies working memory, respondents who saw neutral photos reported less neutral affect when their working memory was depleted than when it was not. If neutral states were merely feeling nothing, then one would not expect neutral feelings to be influenced by altering mental capacity because there would be no experience to alter. A key implication of this work is that neutral states are felt experiences that need cognitive resources to be sustained.

Additionally, it is important to point out that neutral affect is distinct from other nonvalenced states, such as feeling numb or shocked. Numbness arises when people experience an emotional trauma to help them cope with the pain. Just as people respond to physical pain with bodily numbness, people might respond to psychological pain, such as rejection, with emotional numbness (DeWall and Baumeister, 2006). Neutral affect, which is feeling nothing in particular, should be different from numbness, which is feeling that one cannot respond with emotion. Additionally, shock, which is akin to an extreme form of surprise, arises when an unexpected event happens. Like surprise, shock can be good or bad (e.g., the shock that one is pregnant can be either good or bad depending on the circumstances). Feeling nothing in particular (i.e., neutrality) should differ from shock. Gallegos and Gasper (2018) examined whether neutral affect was distinct from numbness and shock by investigating whether experiences of interpersonal rejection produced numbness, shock, or neutral reactions. They found that compared to a control condition, rejection resulted in people feeling numb (i.e., numb, unfeeling, detached, insensitive, and emotionally dead, Cohen’s *d* = 0.33, 95% CI [0.16, 0.49]) and shocked (i.e., stunned, shocked, dumbfounded, astonished, blown-away, all three experiments, *d* = 0.80, 95% CI [0.62, 0.97]), but did not alter neutral affect (i.e., indifferent, nothing, emotionless, so-so, do not feel strongly one way or the other, and meh, *d* = 0.06, 95% CI [−0.11, 0.22]). This is an important finding, in that researchers should be aware that states that have typically been described as nonvalenced can be distinguished from each another. To group these states together can result in perhaps incorrect conclusions, such as when researchers claim that rejection produces neutral reactions (Blackhart et al., 2009).

In sum, the belief that questions the existence or occurrence of neutral affect might stem from (1) an inaccurate definition that neutral affect is feeling literally nothing, (2) the misconception that neutral affect cannot co-occur with other valenced states, (3) the false assumption that English lacks the vocabulary to directly describe the state of feeling neutral, and (4) cultural differences in expressing emotionality. To demonstrate that these assumptions about neutral affect are unwarranted, we reviewed research indicating that (1) people do feel neutral and the intensity of neutral affect varies, (2) feeling neutral is a felt experience that requires cognitive resources, and (3) neutral affect co-occurs with other affects and is different from other nonvalenced states such as numbness and shock.

## Belief: Neutrality is not an Affective State Because Affect Must be Positively or Negatively Valenced

When discussing affective valence, it is important to be clear how one is using the term. According to Colombetti (2005), among other things, valence could refer to either evaluative valence or affective valence. Evaluative valence refers to how the environment is appraised. For example, a person might appraise the situation as dire, resulting in fear. Affective valence refers to the hedonic quality of the emotional state. For example, fear might be experienced as negative if it prevents a person from giving an effective talk, but it might be experienced as positive if it functions as a motivator. Here, we discuss neutrality both in terms of evaluative and affective valence.

### Evaluative Valence

Affect, by definition, is evaluative. The emotional system helps people evaluate the biological significance of the stimuli that they encounter (LeDoux, 1989). Valence is an important component of affect (Russell, 2003; Peters et al., 2006), in that, among other things, affective valence provides critical information concerning whether the environment is experienced as good or bad. Watson et al. (1999) wrote, “Indeed, valence is such a salient aspect of our appraisal process that humans almost instantaneously evaluate their ongoing state as either pleasant/positive or unpleasant/negative…” (p. 828). Evolutionarily, this hypothesis makes sense in that people need to know if something is a threat or an opportunity so that they can avoid or approach it (Nesse, 2004).

Yet, when thinking about the role of valence within affective research, it is worth keeping in mind the distinction between affect and emotion. Emotions operate as a signaling system, grabbing and diverting attention to what is important (Simon, 1967). Valence is such a key feature of emotions that Ortony and Turner (1990) wrote, “… we assume that being affectively valenced is a necessary condition for a state to be an emotion. Excluded from this view is the possibility that an emotion could be affectively neutral” (p. 317). Similarly, Nesse (2004) wrote, “If a situation contains neither threats nor opportunities it will have no influence on fitness. This is why there are few, if any, neutral emotions” (p. 1338). Thus, some researchers argue that emotions must be valenced because a chief function of emotion is to alert people to potentially important threats and opportunities. Because neutral information is probably not of critical importance, some researchers think that emotions cannot be neutral.

Even though it is possible that *emotions* might not be neutral, we argue that *affect* can be neutral. Emotions operate as an urgent signaling device. Because it is typically not critical to know that a situation is neutral, we acknowledge that neutral emotions might be less likely to occur. But, at this point, we would not go as far as to say that neutral emotions do not exist. For example, neutral emotions might arise in situations where it is critically important to attend to neutrality, such as when one is trying to be fair and impartial. However, we believe that it is problematic if researchers mistakenly extend this reasoning about the possibility of neutral *emotions* to neutral *affect*. Specifically, we think that it is a mistake to assume that because affect provides valenced information about the environment, affect cannot be neutral.

We argue that when people evaluate their environments, they not only want to know what is helpful or hurtful, but also what is neither. People’s attention is finite, so just as it is arguably functional to know what is good or bad, it is also functional to know what is neither. It seems like it would be of paramount importance to know what one does not have to concern themselves with. Thus, neutral affect provides information about valence, in that it signals where immediate attention is not needed. Consistent with this view, when discussing how to think about valence, Higgins (2014) wrote “… the nature of valence depended on how neutrality was determined: to understand valence, you need to understand ‘0’” (p. 429; “0” refers to neutrality). Yet, researchers rarely discuss neutrality as a key element of valence. When assessing neutral affect, neutrality is often merely a point or a small region along a single bipolar or two unipolar valenced dimensions (see Cacioppo et al., 1999; Russell, 2003; Larsen et al., 2009). Neutral affect is rarely conceptualized or assessed independently of positive and negative affect. We think that this approach is problematic. Carver and Scheier (1990) nicely illustrated our concerns when they argued that the absence of a state does not mean the presence of another. They said:

…knowing a person is not depressed does not make it reasonable to infer that the person is happy. Knowing a person is not happy does not make it reasonable to infer that the person feels bad. Sometimes, people are affectively neutral (Carver and Scheier, 1990, p. 27).

Similarly, we argue that neutral affect cannot be inferred by the absence of other affects. It should be assessed separately from positive and negative affect. Furthermore, when this is done, neutral affect appears to be a dimension that is somewhat independent of positive and negative affect.

What is the evidence that measures of neutral affect capture unique information that measures of positive and negative affect do not? Recall, we defined neutral affect as the presence of neutral affect, not the absence of positive and negative affect. This definition allows for the possibility that neutral reactions might arise independently of and co-occur with positive and/or negative reactions. It is insufficient to infer neutral affect from the lack of positive and negative affect because neutrality might arise when both are present. Consistent with this view, in studies that examine both evaluative valence and affective valence, neutral affect co-occurs with positive and negative affect (Gasper and Hackenbracht, 2015; Gasper and Danube, 2016; Samson et al., 2016; Gallegos and Gasper, 2018). Moreover, people’s self-reports of neutral affect are not highly correlated with reports of positive and negative affect (Gasper and Hackenbracht, 2015; Gasper and Danube, 2016), suggesting that neutral affect is not simply a state that increases when positive/negative affect decreases. Also, in contrast to the hypothesis that the presence of positive or negative affect implies less neutral affect, sometimes these correlations are positive (Gasper and Hackenbracht, 2015; Gasper and Danube, 2016; Gallegos and Gasper, 2018). Factor analyses of affective states reported in five different samples revealed that in addition to positive and negative affect factors, there was a clear and consistent third, neutral affect factor (Gasper and Danube, 2016). Scatter plot data also revealed that some participants report feeling strongly negative, for example, and neutral at the same time (Hu and Gasper, 2019, submitted). Thus, neutral affect might be an independent dimension, one that provides valence-relevant information that cannot be captured by merely assessing positive and negative affect.

In sum, we believe that it might be a mistake to only consider whether stimuli are evaluated as positive or negative. Valence provides people with information that can inform their actions. Thus, it seems important to know what is good, what is bad, and what is neither. Given people’s finite cognitive capacity, humans not only need a way to prioritize what is important, but also to be aware of what is not as important. A key implication here is that researchers should consider expanding their view of valence to include evaluations of stimuli as good, bad, and neutral. Indeed, neutrality ratings can occur independently of positivity and negativity ratings. Therefore, we argue that neutral affect is indeed affect because it provides important, valence-relevant information.

### Affective Valence

So far, we have focused on evaluative valence, but what about the hedonic experience of neutral affect? Do people experience neutral affect as neutral, pleasant, or unpleasant? Whether a particular affective state is experienced as hedonically pleasurable or painful depends on the context (Barrett et al., 2007, 2011; Condon et al., 2014). For example, even though happiness is typically thought to be hedonically pleasurable, it need not be (Condon et al., 2014). Given this view, how neutral affect is experienced also could be a matter of intentional focus and interpretation, depending on one’s emotional goal and the context in which neutrality arises (Barrett et al., 2007). Below, we review a few ways in which researchers have conceptualized neutral affect with regard to its hedonic valence.

In adaption theories, neutrality reflects people’s current adaptation level, in that it is the state that arises when people have adapted to their environment. Adaptation is experienced as neither good nor bad. It serves as a reference point to evaluate other states (Helson, 1964). Enjoyment, for example, does not come from trying to be neutral, but rather from “… disparity between stimulation and prevailing adaptation level” (Helson, 1964, p. 49). Without some type of neutral state, it would be hard to know what joy and sorrow are like for there is no state to compare them to (Lyubomirsky, 2011). Within this view, neutrality is not a hedonically sought out state, but rather the current standard used to evaluate whether an event increases or decreases pleasure or pain.

Just as neutrality can be used as a standard by which to evaluate the hedonic qualities of other affective states, other affective states can potentially determine the hedonic qualities of a neutral state. Neutrality might be experienced as pleasant or unpleasant depending on whether one is experiencing a decrease in pain or pleasure. For instance, going from a painful experience to a neutral one probably feels like an improvement, whereas going from a joyous state to a neutral one might be experienced as a decline. Thus, neutral affect could be either sought out or avoided.

The idea that a situation could alter how seemingly neutral situations are experienced is evident within regulatory focus theory. One might argue that the *status quo* is a neutral experience – nothing has changed, therefore there should be a neutral response. Yet, the meaning of the *status quo* also depends on the situation. In regulatory focus theory, which discusses people’s affective reactions to gains and losses, achieving the *status quo* (neither a gain nor a loss) need not be experienced as neutral (Brendl and Higgins, 1996; Higgins, 2014; Higgins and Liberman, 2018). Instead, how the *status quo* is experienced depends on whether the person is promotion focused (focused on obtaining gains) or prevention focused (focused on avoiding losses) and if they experience a non-gain or a non-loss. When a person is promotion focused, they are focused on obtaining something. If they do not gain anything (a non-gain), then they experience the *status quo* as disappointment because it reflects not getting the gain that they desired. Conversely, when a person is prevention focused, they want to maintain the *status quo* rather than experience a loss. If they do not experience a loss (a non-loss), then they experience the *status quo* as relief because no loss occurred. Thus, *status quo*, which some might view as producing a neutral state because no change in circumstance occurred, could be experienced as disappointment or relief, depending on whether a person is promotion or prevention focused at that point in time.

In addition, people’s personal and culturally based theories about the desirability of neutral feelings might shape whether neutral affect is experienced as pleasurable or painful. For instance, websites like Reddit contain questions about what neutral affect is like^2^ and whether it is normal^3^. If one’s society values feeling positive, then feeling neutral might be experienced as non-normative and hence a negative state relative to the presumed positive norm. However, if one’s society values feeling balanced and not overly positive, then neutral affect might be experienced as normative and hence an appropriate reaction. For example, a key element within Buddhism is to practice meditation so that people become aware of their experiences. In Buddhism, people who do not attend to their feelings typically respond to neutral feelings by ignoring them. Buddhist philosophy argues that when people ignore their neutral feelings, they are more likely to experience boredom and ignorance because all feelings, including neutral feelings, should to be attended to (Bodhi, 2000; Kudesia and Nyima, 2015). Dhammadinna, a nun, stated that when people have neutral feelings, ignoring them and not knowing can be painful and unpleasurable, whereas knowing the neutral feeling is pleasant (Anālayo, 2017). Thich Nhat Hanh, a Vietnamese Buddhist monk, went even further saying the following about the practice of mindfulness with regard to neutral feelings:

In the process of practicing we discover that the neutral feelings are very interesting. As when we sit, there is a sensation that is neutral. When we bring mindfulness to the neutral feeling, you find that it is quite nice. You see that you already have enough conditions for happiness with a neutral feeling. If you look deeply at the neutral feeling you see that it is wonderful. When you see your feelings passing by like a river, you see that 80% of your neutral feelings are quite pleasant. With mindfulness, our neutral feeling is transformed into happiness (Thich, 2011).

Within this view, awareness of and knowledge about neutral feelings, not the feelings themselves, can contribute to happiness and pleasure.

In sum, we argue that neutral feelings can be hedonically experienced as positive, negative, or neutral. This assertion does not mean that neutral affect does not exist, because we similarly argue, for example, that happiness need not feel positive and fear need not feel negative (Condon et al., 2014). How feelings are experienced depends on the person and context. Neutrality can be a state people use as a reference point, seek out, avoid, or focus on to gain awareness. Neutral states need not be experienced as neutral, but this conclusion does not negate their validity and importance within the affective realm.

## Belief: Neutral Affect is Unimportant Because it Does not Influence Cognition or Behavior

Affective states influence behavior. According to the affect-as-information perspective, this might occur because affective states provide people with information or feedback, which can influence how people think or act (for reviews see Clore et al., 2001; Schwarz and Clore, 2003; Gasper and Isbell, 2007; Gasper and Spencer, 2018). Some researchers might believe that neutral affect is not very important because they assume that neutral states are affect free and hence provide little information or feedback. For instance, Cohen and Andrade (2004) discussed neutral affect as being less informative in the evaluative process than other moods. Neutral affect also is used as a control condition, presumably because no affect is present to alter the experiment (Gasper, 2018). These assumptions, however, might be unwarranted. We argue that, like other affective states, neutral affect can influence cognition and behavior because it too provides people with valuable affective information.

So, what type of affective information might neutral affect provide? One key piece of information that neutral states might provide is that they signal one need not attend to the environment because there is nothing particularly noteworthy in it. This view is reflected in a variety of models. For instance, in Buddhism, there are three main modes of feeling: pleasant, painful, and neutral (e.g., De Silva, 1995). Kudesia and Nyima (2015) argued that these pleasant, aversive, and neutral feelings respectively produce “…action tendencies to prolong pleasant perceptions, remove unpleasant perceptions, and disregard the neutral perceptions” (p. 916). That is, because neutral feelings are neither pleasant nor painful, they are often not noticed and people disregard or remain ignorant of these reactions (Bodhi, 2000; Kudesia and Nyima, 2015). Neutral affect signals that the situation does not need to be attended to because it is not noteworthy or important. A similar view occurs within the core affect perspective (Russell, 2003), in that neutral affect might not be consciously experienced, in part, because it fades into the background due to a lack of a need to attend to it.

In addition to neutral affect perhaps signaling that one does not need to attend to the environment, neutral affect also might signal that one understands the environment. In the AREA model of affective adaptation, Wilson and Gilbert (2008) argued:

People analyze incoming information with two questions in mind: “Is it important to me?” and, “Do I understand it sufficiently?” If the event is deemed to be both self-relevant and unexplained, people allocate attention to it, and the event triggers an affective reaction. Conversely, if the event is deemed to be either unimportant or sufficiently explained, people do not allocate attention to it, and the event does not trigger an affective reaction (Wilson and Gilbert, 2008, p. 372).

Thus, like the Buddhist perspective, this view implies that neutral affect might indicate that the event is unimportant. But it also builds upon the Buddhist view, in that neutral affect might signal that one understands, knows, or comprehends the event.

In addition to signaling understanding, neutral affect might signal that a situation is normal. Lyubomirsky and colleagues’ view of hedonic adaption argues that adaptation occurs when people’s perceptions of something as positive or negative become neutral (Lyubomirsky, 2011; Armenta et al., 2014). Events that are hard to adapt to are those that grab attention, are varied/dynamic, and are novel/surprising, suggesting that neutral events do not grab attention, are not dynamic, and are normative. Brendl and Higgins (1996) also discussed neutrality as potentially reflecting the norm. They wrote, “… normal events should not be perceived as causal, and should therefore be neither supportive nor unsupportive of a goal; that is, they should be appraised as neutral in valence and quality” (Brendl and Higgins, 1996, p. 128). Specifically, using norm theory (Kahneman and Miller, 1986), Brendl and Higgins (1996) argued that objects, attitudes, and events that exemplify the norm produce neutral reactions. Moreover, because the norm easily comes to mind, they also argued that the ease with which knowledge comes to mind might serve as a neutrality cue. For instance, they discussed a study by Ostrom and Upshaw (1968) that examined how easy/difficult it was for people to write down a belief that reflected the various points on an attitudinal scale. Consistent with the notion that neutral affect might reflect something that is easily understood, respondents found it easiest to think of the midpoints and the endpoints. Thus, neutral states might signal that the situation does not need to be attended to because it is not noteworthy, is understood, and is normal.

Lastly, neutrality also might signal that one does not feel one way or the other about the environment. This experience could have a range of potential consequences, including helping people cope. This possibility is nicely illustrated in a parable from another Eastern philosophy/religion, Taoism. In the parable, a farmer’s horse runs away. Neighbors comment that this is bad luck, but the farmer replies “maybe.” The next day, the horse comes back with three other wild horses. The neighbors comment about how wonderful this new development is. The farmer replies “maybe.” The farmer’s son then breaks a leg while trying to ride the wild horse. The neighbors offer sympathy for this negative event. In response, the farmer replies “maybe.” The story continues, but the critical message within it is that the farmer realizes that we can never really know if the situation will be good or bad. The farmer takes a neutral perspective, because valence is yet to be determined. The story reflects the view that sometimes it is important to be impartial and nonjudgmental.

Currently, there are not many studies that empirically examine whether neutral states indeed signal that the situation need not be attended to, that one understands it, that it is normative, and that one does not feel one way or the other. Yet, there are a few lines of work which support the hypothesis that neutral affective states can inform cognition and behavior. For example, in Carver and Scheier’s (1998) model of self-regulation, neutral affect appears to provide information about what is worthy of action. In the model, positive affect signals that one is approaching their goal at a rate faster than expected; negative affect that one is approaching it slower than expected; and neutral affect indicates that one is approaching their goal at an appropriate rate. Consequently, one does not need to change their goal-relevant behavior. Neutral affect signals that one should stay the course. The idea that neutral affect encourages people to continue doing what they are doing might stem from it signaling that one does not need to give extra attention to one’s actions, that one has achieved understanding, or that, because one does not feel one way or the other, no adjustments are needed.

A recent study by Gasper and Danube (2016) also supports the hypothesis that neutral affect signals a lack of preference. They hypothesized that neutral affect signals that one does not feel one way or the other about the object of judgment. Consequently, people might rely on their neutral feelings as a basis for their neutral judgments. In six studies, they found that positive affect accounted for the most variance in positive judgments, negative affect accounted for most of the variance in negative judgments, and neutral affect (not positive, negative, or even ambivalent affects) accounted for most of the variance in neutral judgments. Thus, when it comes to evaluating if someone holds a neutral opinion about an issue, people rely on the information provided by their neutral affect above and beyond that provided by these other affective states. Relatedly, Gasper and Danube (2016) found that neutral, but not negative, attitudes were associated with failing to engage in behaviors that people should do, but often do not do (e.g., eating five daily servings of fruit or vegetables).

In addition to potentially providing information that alters cognition, neutral affect has been hypothesized to have important implications for interpersonal behaviors. In the social-constraints model of mood regulation, Erber and Erber (2001) suggested that neutral states are beneficial in certain situations because of the flexibility they offer. That is, people are sometimes motivated to regulate their affective states toward neutrality to meet specific contingencies. For example, Erber et al. (1996) reasoned that neutral states are most desirable in interacting with strangers because it is uncertain whether such interactions will be positive or negative. Accordingly, people who expect to interact with strangers are likely to down-regulate their current moods, regardless of whether their mood is positive or negative. Similarly, De Silva (1976) conceptualized neutrality as a safeguard against the emergence of sentimental attachments. Along these lines, some organizations have asked their employees to keep their affective experiences at work within a relatively neutral range (e.g., Hochschild, 1983; Judge, 1992; Morris and Feldman, 1996), perhaps because of the idea that a neutral demeanor display promotes a rational work environment. Thus, neutral affect might have important interpersonal consequences, with people regulating their affect to be neutral in order to have better interpersonal interactions.

In sum, neutral affective states might provide people with a variety of information, including that the situation does not need to be attended to because it is not noteworthy, that it is understood, that it is normal, and that one does not feel one way or the other. Currently, there is little empirical research supporting these assertions, but what research exists suggests that neutral affect can shape cognition and behavior in ways that are unique from positive and negative affective states.

## New Theoretical Directions

For too long, researchers interested in affect have ignored neutral states. Perhaps they have done so because it is human nature to focus on what seems noticeable rather than what is not. Yet, noticing what is absent is important. For instance, in the *Hound of the Baskervilles,* Sherlock Holmes solved the mystery by noticing that the dogs did not bark. To understand what affect is and how it functions, it is critical to understand what it means to feel nothing in particular. Toward this end, we sought to understand neutral affect by critically examining three potential beliefs that researchers might have about neutral affect. Specifically, we challenged the following beliefs: (1) it is not possible to feel neutral because people are always feeling something, (2) neutral affect is not an affective state because affect must be positively or negatively valenced, and (3) neutral affect is unimportant because it does not influence cognition or behavior. We believe that setting aside the assumptions behind these beliefs will move theory and methods concerning the nature of affect into new directions.

By considering alternatives to the first belief, there are a few critical ways in which research can move forward. First, for some researchers, it might be a key paradigm shift to think of neutral affect not as the absence of affect, but as the presence of neutral affect. If neutral affect is the presence of an experience, it becomes important to understand what that experience is like and how it functions. Neutral affect now becomes a part of the affective realm rather than a non-affective control condition. Consequently, researchers should acknowledge how neutral affect fits into their theoretical framework and the assumptions that they are making about it. Even if a researcher’s model of affect does not include neutral affect, it is important that they at least explain why, instead of simply ignoring the possibility of a neutral state. Considering these issues might result in researchers reframing their questions concerning the nature of affect. For instance, what features do neutral states share with other affective states? What makes neutral affect unique from other affects? Can neutral affect help people cope? Do people differ in their propensity to feel neutral? If so, how might these individual differences shape thought, action, and mental well-being? Additionally, researchers might need to rethink what constitutes an appropriate control condition (see Gasper, 2018), because neutral affect might not always be an appropriate control condition.

Second, if neutral affect exists, it is paramount that researchers develop an appropriate definition of the construct. Once this is established, researchers can work on developing appropriate means to measure it. In this paper, we provided a specific view; however, other researchers might conceptualize neutral affect in different ways (Gasper, 2018; Yih et al., 2019). For instance, a researcher studying emotion regulation might view neutral affect as a person’s baseline state. Others might view neutral affect as akin to low-arousal affective terms, such as boredom, relaxation, or being quiet (Zelenski and Larsen, 2000). If so, it becomes paramount to be clear how one is defining neutral affect. It also is important to conduct experiments to ascertain whether these constructs are equivalent to each other or reflect distinct states. Just like numbness can differ from neutral affect, we suspect that feeling typical or feeling low-arousal affective experiences such as boredom or tranquility can differ from feeling neutral (as defined in this paper). We encourage research on this topic, because it is our hope that such work would shed light on the complex range of experiences that make up people’s daily, perhaps more mundane, affective experiences.

Third, if neutral states co-occur with positive and negative states, how might they do so? One possibility is that more than one state arises, but they stem from different aspects of the same situation. For instance, a person might describe their feelings of eating lunch with their grandmother as both happy and neutral. The happiness stems from the fact that they enjoy their grandmother’s company, whereas the neutrality might reflect the fact that the food was unremarkable. Both states are felt but refer to different aspects of the experience. Interesting questions arise as to when it is best to focus on each element (Grandma or the food) or when it might be best to focus on the combined affective reaction (lunch with Grandma). A second possibility is that instead of each state stemming from different elements in the same experience, the two states might combine to form an independent, unique, affective experience. For example, happiness and neutral affect might be experienced as feeling happy-go-lucky – the situation is appraised as positive and one has no preferences because all is good. Conversely, a negative and neutral state together might be experienced as apathy – the situation is appraised as negative and one has no preferences because it is all bad. A third possibility is to also think about whether neutral states in combination with other states might produce ambivalent reactions. Ambivalence is often thought about in terms of feeling positive and negative at the same time. However, it might be that people experience ambivalence when they feel positive or negative affect with neutral affect Hu and Gasper, 2019, submitted. For example, ambivalence might arise when thinking about lunch with Grandma because it was both positive and neutral. If so, what are the ramifications for how this type of ambivalence plays out to influence thought, action, and psychological well-being?

The second belief we focused on concerned the notion that affect must be valenced. We discussed this belief both in terms of evaluative valence and affective valence. In terms of evaluative valence, a range of questions arise. For instance, do people evaluate novel stimuli not only in terms of whether the stimuli are positive or negative, but also neutral? If positively valenced feelings typically signal approach, and negatively valenced feelings avoidance, do neutral feelings necessarily result in neither? Or is it possible, as in theories like the evaluative space model, that a positivity offset exists in which neutral states promote an approach motivation because otherwise people might miss potential opportunities in their environment (Cacioppo et al., 2012)? If something is evaluated as neutral, how stable or malleable is this experience?

In terms of affective valence, viewing neutral affect as an affective reaction generates many interesting hypotheses concerning the hedonic consequences of feeling neutral. In the United States, happiness is highly valued in that people seek out situations where happiness is likely to occur (Mesquita and Markus, 2004). Indeed, many social contexts in the U.S. promote happiness or positive feelings as their primary purpose, and questions or comments such as “Are you having fun?” and “I’m glad you are happy.” are commonplace (Markus and Kitayama, 1994). One consequence of this promotion of happiness might be that some people are concerned when they experience neutral rather than happy feelings. In fact, research suggests that valuing happiness to an extreme degree in US samples can lead to decreased well-being (Mauss et al., 2011), and depression (Ford et al., 2014), as well as increased loneliness and weakness in social connections (Mauss et al., 2012). People might view their neutral feelings as indicating that they have not obtained the ideal of being a happy person. This focus on feeling happy thus might have some negative psychological consequences. Perhaps people might be psychologically healthier if, instead of focusing on happiness and excitement, they also focus on balance and moderation that might arise with neutral affect. If Thich Nhat Hanh is correct, then paying attention to neutral feelings can lead to happiness. Thus, when it comes to practical ramifications, viewing neutral affect as an important daily state might result in acknowledging a different, and potentially more attainable, path toward mental well-being.

The third belief we discussed was that neutral affect is not important because it does not influence thought or action. By signaling nothing in particular, neutral affect might result in one doing nothing in particular. As we noted, neutral affect potentially provides a range of affective information that could shape thought and action. Neutral affect might indicate that attention is not needed, that one understands the situation, that the situation is normal, and that one does not have feelings one way or the other (i.e., no preference). Research needs to be conducted to test these speculations, but they could have interesting implications. If neutral affect signals that attention is not needed, for example, then neutral affect could potentially influence memory. If people do not attend to neutral stimuli, those stimuli might be less likely to be recalled. In addition, neutral affect might signal understanding and, if so, then people who feel neutral might be more likely to interpret their experiences as indicating that they understand complex, novel, or illogical arguments. If neutral affect signals normality, then neutral affective reactions might serve as indicators of prototypicality, perhaps leading to people being more inclusive in their viewpoints. Lastly, if neutral affect signals no preference, then neutral affect might be an ideal state when impartiality, balance, or fairness is required, such as when serving on a jury. Given the potential information that neutral affect might provide, there are a wealth of questions that future research could address to understand how neutral affect might shape cognition and behavior.

## Coda

Researchers have beliefs about what they investigate. It is important to acknowledge these beliefs, examine the rationale behind them, and empirically test them. In this paper, we challenged three beliefs that researchers might have about neutral affect, specifically, that neutral affect does not occur, that affect must be valenced, and that neutral affect is of little importance. In the process, we suggested and provided support for alternative conceptualizations. We acknowledge that research on neutral affective reactions is sparse, but we hope by discussing and dispelling some misconceptions about it that researchers will expand their theorizing, methodology, and practice to include neutral affect. To understand the affective landscape, researchers must pay attention to all states, including what happens when one feels nothing in particular.

## Data Availability Statement

The datasets analyzed in this manuscript are not publicly available. Requests to access the datasets should be directed to KG, kxg20@psu.edu.

## Author Contributions

KG wrote the first draft of the paper and conducted the reanalysis of Gallegos and Gasper, (2018) data. LS and DH revised the first draft. KG, LS, and DH conducted research for the paper, edited the paper, and checked references for accuracy.

### Conflict of Interest

The authors declare that the research was conducted in the absence of any commercial or financial relationships that could be construed as a potential conflict of interest.

## Footnotes

^1^To understand how neutrality could be differentiated from positivity or negativity, one interesting method to consider is the semiotic square, which is used in oppositional analysis (see Corso, 2014).

^2^https://www.reddit.com/r/NoStupidQuestions/comments/2z7cbj/what_is_it_called_when_you_have_no_strong/ for details of the post regarding how it feels like to be neutral.

^3^https://www.reddit.com/r/TooAfraidToAsk/comments/axpu1x/is_it_normalokay_to_almost_always_feel_sort/ for details of the post regarding whether it is normal to feel neutral.
